# On automated RBAC assessment by constructing a centralized perspective for microservice mesh

**DOI:** 10.7717/peerj-cs.376

**Published:** 2021-02-01

**Authors:** Dipta Das, Andrew Walker, Vincent Bushong, Jan Svacina, Tomas Cerny, Vashek Matyas

**Affiliations:** 1 Department of Computer Science, Baylor University, Waco, TX, USA; 2 Faculty of Informatics, Masaryk University, Brno, Czech Republic

**Keywords:** Microservices, REST, RBAC, Access control, Authorization, Security, Static code analysis, Systematic architecture reconstruction

## Abstract

It is important in software development to enforce proper restrictions on protected services and resources. Typically software services can be accessed through REST API endpoints where restrictions can be applied using the Role-Based Access Control (RBAC) model. However, RBAC policies can be inconsistent across services, and they require proper assessment. Currently, developers use penetration testing, which is a costly and cumbersome process for a large number of APIs. In addition, modern applications are split into individual microservices and lack a unified view in order to carry out automated RBAC assessment. Often, the process of constructing a centralized perspective of an application is done using Systematic Architecture Reconstruction (SAR). This article presents a novel approach to automated SAR to construct a centralized perspective for a microservice mesh based on their REST communication pattern. We utilize the generated views from SAR to propose an automated way to find RBAC inconsistencies.

## Introduction

With the software industry’s growth, the complexity of security administration is becoming more and more challenging. As the current software development trend is moving rapidly from monolithic to MicroService Architecture (MSA), we must address communication patterns not only for the simple client to server scenarios but also for service to service scenarios. Since the client-server communication pattern has existed for many years, its security implications have already been well addressed. In contrast, not much has been studied for service-to-service communication patterns.

Currently, the most popular way to establish communication between services is to use Representational State Transfer (REST) (Vural, Koyuncu & Guney, 2017; Salah et al., 2016). Developing a secured REST-based infrastructure is relatively easy for a smaller number of microservices. However, the security aspects gradually become more complex as the number of microservices grows. Due to their high feature set and operational complexity, modern microservice-based applications tend to have a large number of individual microservices developed separately by separate teams. Enforcing a robust security solution for such applications is tedious for developers and might lead to security disagreement among microservices. This is because individual developers only have an idea of a subset of microservices they maintain but lack an understanding of the overall picture. Even system architects may not understand the complete picture of the application since many of those microservices may not be in the initial blueprint of the application but rather were added later.

Thus, we need to establish an automatic way to generate the overall communication pattern for the whole application before diving into the security aspects. This is done through a process of Systematic Architecture Reconstruction (SAR) in which overall views are constructed from existing application artifacts. SAR is divided into four phases: extraction, construction, manipulation and analysis.

In this article, we first introduce a solution for automatic SAR of a microservice application, which generates a view of the microservices’ REST communication pattern. By automating the first three phases of SAR and utilizing the constructed views, we can focus on the analysis phase and present an approach to enumerate possible security loopholes in the application. More specifically, we focused on finding Role-Based Access Control (RBAC) inconsistencies among microservices using static code analysis. We present a case study on a single enterprise application called Teacher Management System (TMS) consisting of four individual microservices. This application was developed separately but re-purposed here as a testbed for performing static code analysis. Our work focuses on intra-and inter-microservice inconsistencies highlighting all possible RBAC issues.

An application’s core security requirement is to ensure that it can only be used by legitimate users (Mohanty, Mohanty & Balakrishnan, 2016). RBAC is one of the popular methods of securing REST services where each user of the application is assigned to a set of roles that grant access to different parts of the system. In microservice-based applications, there can be two different abstractions to enforce RBAC rules. First, centralized among all the microservices and, second, per microservice-based.

Thus, next in this article, we focus on the centralized approach. Finding inconsistencies among RBAC rules in a large system is a cumbersome and difficult task due to different levels of abstractions, poor coding practices, and coupling with third-party services. According to a survey conducted in 2014 by the International Data Group (Mohanty, Mohanty & Balakrishnan, 2016), about 63% of applications have not been tested for security vulnerabilities. This can be easily mitigated by enforcing standard security features during the regular software development process (McGraw, 2004). Ignoring such security vulnerabilities is expensive. Security breaches can cost companies billions and require significant time and effort to resolve. For example, the 2014 eBay hack, which was caused by improper access control restrictions, impacted over 145 million users (Swinhoe, 2020). Being able to list possible security vulnerabilities automatically can significantly reduce the likelihood of such incidents.

System administrators should wisely choose the approaches to test the security vulnerability of the system. The most accurate outcome from such a test can be obtained via rigorous penetration testing. However, such an approach needs the application to be fully deployed, and running penetration tests against a production deployment could lead to disruption for end users. Also, it is difficult to emulate all possible scenarios for penetration testing. In contrast, static code analysis can be a much easier alternative that does not require an application to be deployed and hence is more cost-effective. Although static code analysis is no panacea, when carefully implemented, it can detect many vulnerabilities. It is for these reasons we use static code analysis for our automated SAR process.

The article is organized as follows. Section two discusses the related work and state of the art. “Proposed Method” describes our proposed method in detail, and section four explores a case study. Finally, we conclude our article by summarizing our work outcomes, describing our future goals, and listing the references. Throughout the article, the terms “inconsistency”, “violation” and “issue” are used interchangeably to indicate a potential flaw.

## Related work

In this section, we present related work from the two different perspectives considered in this article. First, we assess the limitations of RBAC analysis in the context of enterprise systems. Next, we assess existing approaches for the SAR.

### Role-based access control

In microservice-based applications, each microservice implements a subset of features. End-users or other microservices can access these features through an application programming interface (API). There are typically two main API development choices: REST and Simple Object Access Protocol (SOAP) (Tihomirovs & Grabis, 2016). While REST is an architecture for API development that works over standard HTTP protocol, SOAP is just a protocol. For many years, SOAP was a standard approach for web service interfaces. However, it has been dominated by REST in recent years. According to Stormpath, over 70% of public APIs are designed using REST (Hunsaker, 2015). The main advantage of REST compared to SOAP is its simplicity and ease of learning. REST is lightweight and hence better suited for a wide range of devices, including mobile devices (Wagh & Thool, 2012). Apart from that, REST uses JavaScript Object Notation format which is faster to parse compare to Extensible Markup Language used in SOAP (Tihomirovs & Grabis, 2016; Castillo et al., 2011).

Securing REST API endpoints is generally easy when existing HTTP security approaches are leveraged instead of implementing a new security model (Sudhakar, 2011). Securing REST endpoints involves both authentication and authorization (Brachmann, Dittmann & Schubert, 2012). Authentication is the process of verifying the credentials associated with a particular request. Different enterprise applications use different strategies to authenticate incoming requests, such as basic authentication, token-based authentication, hash-based digest authentication, OAuth, etc. (Lee, Jo & Kim, 2015). On the other hand, authorization involves verifying whether a request connection is allowed to perform a particular action through a REST endpoint. Mandatory access control, discretionary access control, attribute-based access control and RBAC are popular approaches for enforcing authorization (Sandhu & Samarati, 1994). In this article, instead of authentication breaches, we focus on exploring and detecting possible authorization inconsistencies, specifically role-based authorization inconsistencies.

Role-Based Access Control has been widely adopted as an alternative to classical discretionary and mandatory access controls because of its advancement in flexibility and detail of control (Sandhu & Samarati, 1994; Sandhu et al., 1996). It regulates users’ access to information and system resources based on activities that users need to execute in the system and requires the identification of roles in the system (Ahn & Sandhu, 2000). RBAC’s administrative capabilities have made it stand out from the alternative approaches because system administrators can statically or dynamically regulate user’s access by defining roles, role hierarchies, relationships, and constraints (Ferraiolo, Cugini & Kuhn, 1995). For distributed systems, another advantage is that RBAC administrative responsibilities can be divided into central and local protection domains (Ferraiolo, Cugini & Kuhn, 1995). In the case of microservice-based applications, these can be translated into central policies for all associated microservices and per microservice-based policies. Central RBAC policies can be enforced by delegating authentication and authorization tasks to a separate identity management tool, such as Red Hat’s Keycloak (Red Hat Inc, 2020a). On the other hand, individual microservices can carry out such policies using security features of underlying frameworks, such as spring-security for spring-based applications (Scarioni & Nardone, 2019).

Due to the high impact of security-related issues, much research and development have been done addressing role violations. Ciuciu, Tang & Meersman (2012) described one such strategy where appropriate security annotations are recommended for developers based on the ontology extracted from the business information. However, since this recommendation strategy works only based on business information irrespective of source code, if the business information provided is flawed, then the recommendation will also be faulty.

One similar study focused on finding security vulnerabilities of API implementations among different libraries based on security-sensitive events (Srivastava et al., 2011). It finds discrepancies among security policies associated with the same API using a flow graph. The inherent drawback of this approach is that it requires multiple independent implementations of the same API, and it can not find which ones of whose multiple implementations are faulty. Another research study described a model-based approach for testing access control rules based on consistency, completeness and redundancy (Xu et al., 2012). It checks whether access control rules are consistent across the methods, whether they are unnecessarily repeated, and whether they covered all subset of permissions. However, the coverage of access control rules over a set of methods does not necessarily relate to security issues. In Xu et al. (2012), the system under study does not allow a user to rent a book on maintenance due to the incompleteness of access control rules, which is more of a system flaw rather than a security issue. In contrast, our proposed method finds whether a user can access a resource-restricted by one RBAC rule through an alternative path that has less restriction.

The tool FixMeUp (Son, Mckinley & Shmatikov, 2013) proposed an automated way to fix access control issues in PHP applications using static code analysis. It automatically edits the source code to resolve access control issues. Although it seems compelling to automate the task, it might lead to syntax errors and might result in unintended consequences in case of false positives. On the other hand, our RBAC tool pinpoints the location of possible inconsistencies in the source code without adversely affecting the codebase since it does not modify the source code while performing analysis.

The most similar analysis to our proposed method has been described by Walker et al. (2020). That tool performs static code analysis on enterprise JAVA applications to find issues in RBAC rules defined using security annotations. The key difference is that it only considers intra-microservice issues, while our method works for both intra- and inter-microservice issues, taking into account all the microservices that constitute the application.

Freudenthal et al. (2002) proposed a distributed RBAC (dRBAC) mechanism that decentralizes the trust-management across multiple administrative domains. Due to its distributive nature dRBAC is highly scalable for a large number of mutually anonymous users. It features third-party delegation that enables one authorized entity to entrust roles created by another entity. Besides, it controls the access levels for the same resource by valued attributes. Also, dRBAC presents continuous monitoring by utilizing a pub-sub model to ensure the validity of trust relationships for extended interactions. In this article we do not assess such decentralized RBAC techniques, rather we assume that the user authentication and role mapping are handled through a centralized service while individual microservices are responsible for the imposition of those roles on API endpoints.

Separation of Duties (SoD) has been widely studied in the context of RBAC. It ensures data integrity and fraud prevention by distributing critical tasks among multiple users (Basin, Burri & Karjoth, 2009). It enforces that no single user can execute all actions and thus any kind of fraudulent activity will cause collision among at least two users (Habib et al., 2014). In RBAC, SoD can either static or dynamic (Sandhu, 1990). In the static separation of duties (SSD) constraints are enforced during the assignment of users to roles. On the other hand, in dynamic separation of duties (DSD) constraints are activated on the roles within a user session (Omicini, Ricci & Viroli, 2005). In this article, we are not considering the user assignments and user sessions. Instead, we performed static code analysis that solely focused on a subset of SSD including statically defined roles and role hierarchies.

### Software architecture reconstruction

Although many studies address access control issues, most of them are focused on single microservice or monolith applications. However, modern cloud-based applications are commonly designed as a set of microservices for better flexibility and scalability (Salah et al., 2016). The key challenge to perform a holistic analysis across multiple microservices is the automated construction of the application’s centralized perspective. SAR extracts a representation of software architecture from source code or documentation through an iterative reverse engineering process (Bass, Clements & Kazman, 2003). It is historically defined with four phases: extraction, construction, manipulation and analysis (Bass, Clements & Kazman, 2003). In the extraction phase, all necessary artifacts are collected. Each set of related artifacts is relevant to a *view* that represents relations among certain elements of the software architecture (Bass, Clements & Kazman, 2003). The construction phase creates canonical representations of the views. Then the manipulation phase combines the views to create a more compact representation to answer specific questions in the analysis phase. Lastly, the analysis phase answers specific research questions from the reconstructed views. In this article, the analysis phase addresses the detection of possible RBAC inconsistencies. Also, to the best of our knowledge SAR has not been used to detect RBAC inconsistencies in MSA.

One approach of SAR of microservice-based systems is described by Rademacher, Sachweh & Zündorf (2020). This method describes different modeling based on different viewpoints (Rademacher et al., 2020) where domain modeling is based on bounded context, services modeling is based on REST calls, and operation modeling is based on deployment specifications, for example, Dockerfiles.

The Model-Driven Engineering (MDE) approach is commonly used in SAR. In MDE, models are used as first-class entities to depict an efficient representation of the software architecture (Cicchetti et al., 2013). Alshuqayran, Ali & Evans (2018) described a manual analysis through the MDE approach to reconstruct the architecture of microservice-based open-source projects. They defined a metamodel which is then mapped to the architecture using mapping rules. The metamodel and mapping rules are initially created for one system and then refined and validated using seven additional systems. However, the authors did not apply their reconstruction strategy to answer specific questions.

Ibrahim, Bozhinoski & Pretschner (2019) proposes an approach to derive MSA module topology from container-based deployment configuration files, more specifically, from Docker Compose files. In addition to topology, they extracted the *attack graph*, a directed acyclic graph, to identify attack paths that lead to vulnerability exploitation. Their implementation is based on a open-source vulnerability scanner for Docker containers named Clair (Quay, 2020).

The MicroART tool described by Granchelli et al. (2017) extracts the deployment architecture of a microservice-based system from the source code repository. It utilizes a domain-specific language to represent key elements of the architecture by using the MDE approach. It employs runtime log analysis to discover containers, network interfaces, and service interactions. However, users need to provide a reference to the container engine since MicroART does not automatically detect it from deployment configuration files.

Our proposed method reconstructs MSA architecture based on the REST communication pattern, similar to the service modeling described by Rademacher, Sachweh & Zündorf (2020). However, unlike that system, which depends on a Service Modeling Language (Rademacher et al., 2020), our reconstruction is solely based on static code analysis and works independently.

## Proposed method

Enterprise applications are typically organized into a three-layer structure: controller layer, service layer, and repository layer. It is also common to organize microservices into the presentation layer, business layer, persistence layer, and database layer (Richards, 2015). These two commonly used structures essentially indicate the same strategy. The presentation layer maps to the controller layer, which defines API endpoints, and the business layer maps to the service layer, which contains business logic. The persistence layer maintains data access objects to interact with the database layer (Alur et al., 2003). These two layers (persistence and database) are consolidated into the repository layer in the three-layer architecture (Richards, 2015; Steinegger et al., 2017).

Microservices typically communicate over REST APIs (Salah et al., 2016). Each microservice’s controller layer defines the REST endpoints that serve as request entry points for that particular microservice. Requests are delegated from the controller layer to the service layer. The service layer typically implements business logic. It processes the request and generates an appropriate response. The service layer can also incorporate with the persistence layer to store and retrieve data relevant to a specific request. However, sometimes the service layer depends on other microservices to process the request. In that case, it creates REST calls to other microservices and implements business logic based on the response. This describes a typical REST communication scenario among microservices. In particular, the service layer of one microservice makes REST calls to other microservice’s controller layers to implement its business logic. Thus, the REST endpoints of each microservice can be either accessed by end-users or other microservices.

Enterprise frameworks adopted annotation-based configuration to define REST endpoints, for example, @RestController annotation in Spring-based JAVA applications and @app.route annotation in Flask based Python applications (VMware Inc, 2020; Pallets Projects, 2020). Since the REST endpoints are the entry points to the microservice, securing them is the single most important task for the developers. While there are several ways to enforce role-based authorization, the most widely adopted method in enterprise applications is to define authorization realms through the application server (Oberle et al., 2004) or through separate identity management tools like Keycloak (Red Hat Inc, 2020b). A realm is a security policy domain defined in the application server that contains a collection of users (Jendrock et al., 2014). These users might be further organized into several groups (Jendrock et al., 2014). Centralized authorization systems like Keycloak handles user authentication and role mapping. But such systems do not verify whether developers properly enforced RBAC policies during API implementation or not. For example, some API endpoints might have missing RBAC roles. In that case, any authenticated user can access those endpoints. Similarly, two API endpoints with different roles might eventually access the same entity which might be unintentional and left unnoticed. These inconsistencies are not flagged by the centralized authorization system and thus defining authorization policies are not enough to secure the endpoints. Developers need to enforce those policies within the application’s source code that runs in that application server. Designing proper authorization policies are just one part of ensuring robust RBAC enforcement, we need to consider coding problems that might lead to security loopholes. In this article, we focused on detecting such coding problems through static code analysis. Also, it is important to classify these problems to understand the severity and origin of them. We have defined five types of possible inconsistencies or violations:

1. Missing role violations: this type of violation occurs when an API endpoint does not have any roles associated with it. In this case, all authenticated users can access the endpoint. Such violation typically happens when developers forget to enforce authorization roles on an API endpoint. However, it could be false-positive, for example, some API endpoint might be intentionally left open for all users.
2. Unknown access violations: if an API endpoint contains an authorization role that is not present in the user-defined role hierarchy, then we define it as an unknown access violation. Usually this type of violation results from typographical errors and in most cases, such typos are left unnoticed since they do not cause any compilation errors. As a result, legitimate users with proper access are denied from accessing the endpoint.
3. Entity access violations: if input and output that is, request and response types of two API endpoints are similar but they have different authorization roles, then we classify it as an entity access violation. This kind of violation indicates that the same entity is being accessed by users with different access roles.
4. Conflicting hierarchy violations: this type of violation happens when an intermediate method in the service layer or repository layer contains two different roles that are ancestor of each other’s in the role hierarchy. This violation signifies that users with a junior role are accessing some functionalities that might be intended for users with a senior role (Walker et al., 2020).
5. Unrelated access violations: similar to conflicting hierarchy these violations focus on intermediate methods instead of endpoint methods. When an intermediate method contains two multiple roles that are located in different subtrees of the role hierarchy, we classify it as an unrelated access violation. This type of violation indicates poorly separated concerns while distributing access roles across different functionalities of the application (Walker et al., 2020).

Like REST configurations, authorization policies are typically applied by annotating methods or functions with appropriate security annotations. These annotations can differ based on the framework used to develop the application; for example, JAX-RS security annotations are used with JAVA EE based application (Oracle, 2020). A similar approach to enforce RBAC using annotation can also be found in Python applications based on the Flask framework (Thio, 2020). These security annotations define the level of restrictions applied to the associated methods or functions. Table 1 highlights the most commonly used security annotations in JAVA EE applications supported by JSR 250 (Mordani, 2016; Oracle, 2020).

**Table 1:** JAVA EE security annotations.

Annotation	Description
@permitAll	All security roles are permitted
@denyAll	No security roles are permitted
@rolesAllowed	List of permitted security roles

For example, if we add a @rolesAllowed(ADMIN) annotation to a controller endpoint method, only the users that have the “ADMIN” role (defined in the realms) can access the endpoint. However, since the number of such endpoint methods can be significant and can grow over each iteration of the development cycle, it is possible to introduce inconsistencies among the allowed roles or even missing roles. Moreover, since these inconsistencies and missing roles do not cause any compilation or run time error, it is tempting for developers to overlook them, and that might result in potential security loopholes.

Our proposed method analyzes a set of microservice artifacts that communicate with each other through REST calls. It finds potential RBAC violations for the whole microservice mesh by scrapping security metadata of individual microservices and by combining them based on their REST communication flow. We divided the analyzer into three modules: a discovery module, a flow-matcher module, and an analysis module. The discovery module implements the extraction phase of SAR. It collects endpoint specification and security metadata. Next, the flow-matcher module performs the construction and manipulation phases of SAR by resolving the interaction among microservices. Finally, the analysis module completes the analysis phase of SAR and detects potential RBAC violations based on the other two modules’ output.

The discovery module performs static code analysis on individual microservice artifacts. It detects the REST endpoints and security roles attached to those endpoints. Apart from that, it also lists the REST calls to other microservices, which are typically implemented in the service layer. The discovery module works for both source code artifacts and bytecode artifacts (e.g., JAR file, Python bytecode) and thus provides generalization for both interpreted languages (e.g., JAVA, Python) and compiled languages (e.g., C++, Go). The source code version of the discovery module takes a microservice artifact as input and parse class definitions while the bytecode version does the same using bytecode analysis. As discussed above, both REST endpoints and security policies are typically defined using the annotation-based configuration in enterprise applications. The descriptions of these annotations are well structured and preserved in the source code and in the bytecode. The discovery module scans each class to find REST annotations and security annotations that define REST endpoints and security roles, respectively. It aggregates class-level annotations with method-level annotations to derive the complete definition of each REST endpoints. It collects the allowed roles, port, path, HTTP type, type of request object, and type of response object for each endpoint. It takes account of all standard HTTP types, with the most commonly used ones being *GET, POST, PUT* and *DELETE*. The discovery module then further analyzes service layer classes to detect REST calls to other microservices. For each REST call, it detects the URL, HTTP type, type of request object, and type of response object. It parses REST client definitions to gather those attributes.

However, detecting the URL string involves further intensive analysis since the URL string is usually constructed by performing consecutive append operations in different parts of the source code. For this, our discovery module applies a backward recursive data flow analysis from the point where the URL is used to the point where the URL was initialized. In each intermediate step of the data flow where the URL was referenced, it scans for any append operations and resolves them to restore the final URL. Parts of the URL may also be constructed using values defined in the configuration files instead of hardcoded strings within the source code. Our module also scans configuration files of the project to resolve those values. Finally, the discovery module generates method-call graphs for individual microservices. It takes each controller method as the root node and populates child nodes by traversing subsequent method calls to the service layer and repository layer methods. For each microservice, the discovery module organizes all the scrapped information described above into a usable structure and passes them to the flow matcher module and analysis module.

As discussed by Walker et al. (2020), RBAC security analysis for individual microservices is insufficient. It fails to acknowledge violations when an end-user gains access to a normally restricted resource by creating a proxy request through another microservice mediating the resource access through service layer REST calls. To detect such violations, we need to consider the whole MSA mesh instead of a single MSA, and we need to resolve REST communications between them to construct a complete centralized perspective. In our proposed model, the flow matcher module constructs the centralized communication graph for the whole MSA mesh. It takes descriptions of REST-endpoints and REST-calls for each microservice prepared by the discovery module. It combines all the REST endpoints into a list and all the REST calls into another list. Then it performs a brute force matching between those two lists to resolve all REST communications among the microservices. This involves matching the URL (including port and path), HTTP method, request type, and response type.

However, it is common for modern microservices to use service discovery and service registry instead of a hardcoded IP address in the URL (Montesi & Weber, 2016). To resolve this, our flow matcher module matches both the IP address and service name and checks if one of them matches. The service name is usually defined in each microservices’ configuration files and scrapped during the discovery phase. The flow matcher module also generates a diagram of REST communication for the whole microservice mesh for better visualization.

**Figure 1:**
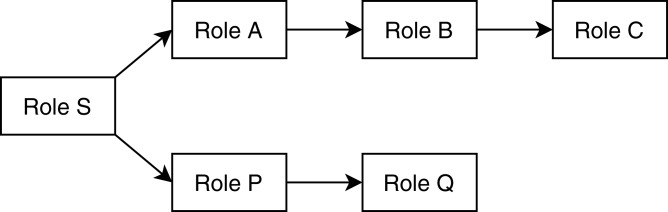
A sample user-defined role hierarchy tree. Senior roles are higher in the tree; in this example, Role S is the most senior role, Role A is senior to B, which is senior to C. Role P is senior to Q.

**Figure 2:**
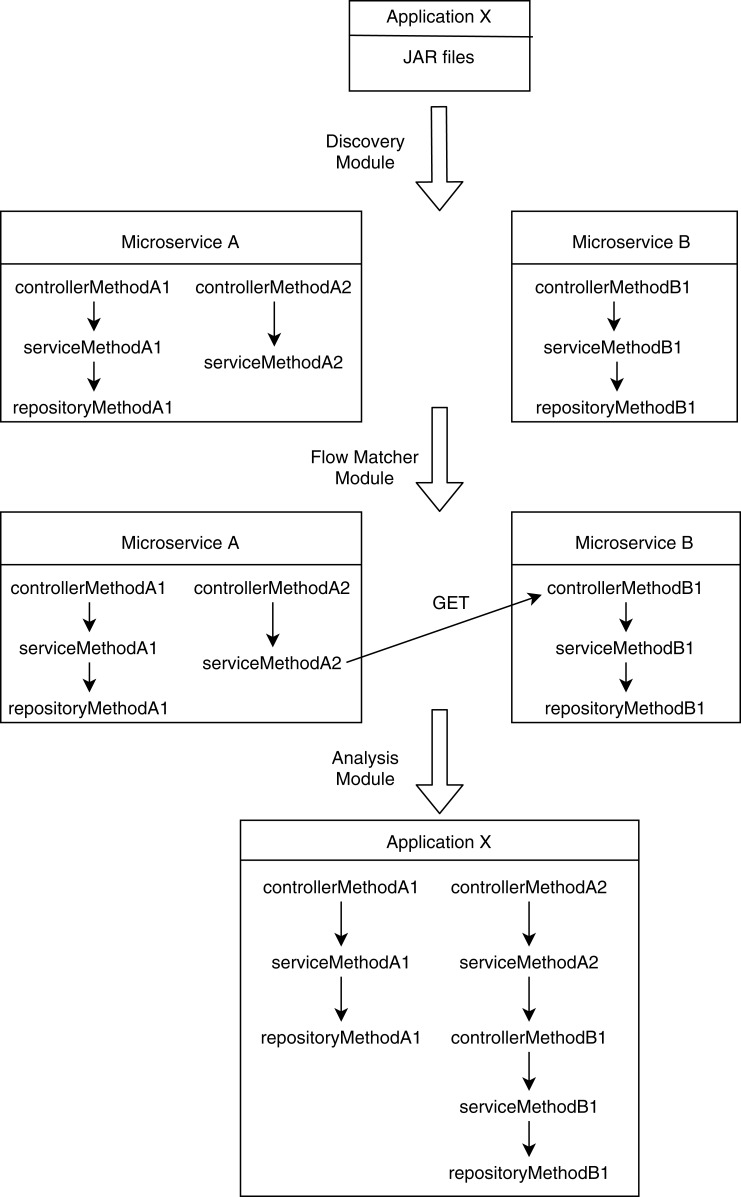
Construction of combined method-call graphs.

The analysis module takes descriptions of method-call graphs and allowed roles from the discovery module and REST communication descriptions from the flow matcher module. Additionally, it takes the role hierarchy tree from the user. Figure 1 shows a user-defined role tree passed to the analysis module as input. Roles higher in the hierarchy tree are senior to the roles below in the tree; senior roles should have all the access rights junior roles have, plus additional rights the junior roles do not have. Roles in separate paths of the hierarchy are not related to each other. The analysis module combines method-call graphs of different microservices based on their REST communication. Figure 2 depicts a typical scenario of how combined method-call graphs are constructed. Each node of the combined graphs can be a controller node, service node, or repository node. Typically, only the controller nodes contain RBAC information, that is, a list of allowed roles; however, the service layer and repository layer nodes can also have RBAC information. To find potential RBAC violations in those layers, the analysis module loops through all the nodes and analyzes the roles associated with them. The first three types of violations are only related to controller nodes. If any controller node does not have any roles associated with it, we detect it as a missing role violation. This is the most common type of violation that might occur since missing roles on controller methods do not cause any compilation errors. If a node contains a role that is not defined in the user-provided role hierarchy, we detect it as an unknown access violation. This type of violations typically results from typographical errors. If request types, response types, and HTTP types of two controller methods are equal, but they have different RBAC roles, we detect it as an entity access violation. This violation implies similar access to a particular entity with different roles.

**Figure 3:**
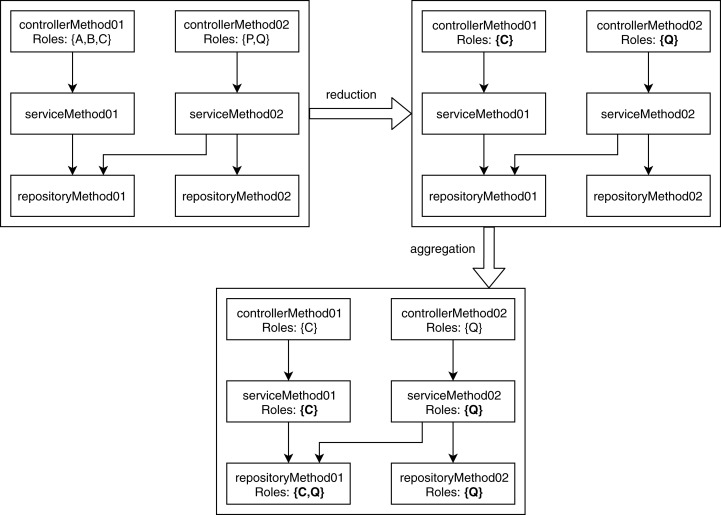
Reduction and aggregation of RBAC roles.

The unrelated access and conflicting hierarchy violations occur when a node contains multiple roles after performing the reduction and aggregation. In the reduction phase, the analysis module goes through each node and keeps the lowest role defined in the user-provided role hierarchy. The significance of this reduction is that it defines the minimum role required to access a specific part of the application. After reduction, in the aggregation phase, the analyzer traverses each graph and copies the allowed role from the parent node to the child node. If a child node contains an RBAC role or a child node has multiple parents with different roles, then it aggregates the roles for that particular child node. Figure 3 shows how the analysis module labels each child node using the RBAC roles of its parent nodes according to the role hierarchy shown in Fig. 1.

The conflicting hierarchy violation occurs when a node code contains two different roles where one role is an ancestor of another role in the user-defined role hierarchy. This violation indicates a place where a junior role potentially accesses an area reserved for a more senior role. It is only a potential violation because it is ambiguous whether a junior role is accessing an area reserved for a senior role or the senior role is accessing something allowed for the junior role (Walker et al., 2020). The unrelated access violation is the opposite of the conflicting role violation. It happens when a node contains two roles located in a different subtree of the user-defined role tree, that is, one role is not an ancestor of another role. This violation indicates areas where unrelated roles are accessing the same application area, which may indicate poorly separated concerns that could be refactored (Walker et al., 2020). For example, considering the role hierarchy shown in Fig. 1, if a node has roles {A, C} then it is detected as a conflicting hierarchy violation, and if a node has roles {A, P} then it is marked as an unrelated access violation. The categorization of violations defined in our proposed method is mostly similar to the ones discussed by Walker et al. (2020). However, Walker et al. (2020) only considered only a single microservice at a time, whereas we also analyze inconsistencies across microservices.

Our system finds potential RBAC violations based on a user-defined role hierarchy for the whole microservice mesh (a set of microservices). It warns the developer about potential violations by providing a report of specific locations where the violations are detected and the categories, as discussed above. While some of the detected violations may be false-positive and intentional, our proposed method provides an overall idea of all possible RBAC violations for a large and complex system. The categorization of the violations helps the developer understand each violation’s severity, while the specific locations of the violations help to find and fix them easily.

## Case study

**Figure 4:**
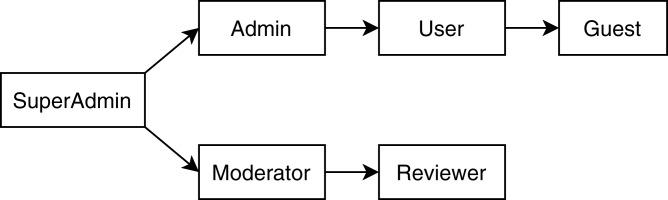
Role hierarchy tree of the TMS application.

The TMS^1^ 1https://github.com/cloudhubs/tms2020. is an enterprise application developed at Baylor University for Central Texas Computational Thinking, Coding, and Tinkering to facilitate the Texas Educator Certification training program. The whole TMS system consists of four individual microservices: user management system (UMS), question management system (QMS), exam management system (EMS) and configuration management system (CMS). All of the microservices are developed using the Spring Boot framework (Walls, 2016) and structured into the controller, service, and repository layers. The RBAC authorization is enforced using annotations on each controller method for the individual microservices, while the central authentication and authorization policies are defined using Keycloak (Red Hat Inc, 2020a). Figure 4 shows the role hierarchy tree for the TMS application. For our case study, we added mutants (Jia & Harman, 2011) for each type of violations that resulted in a total of seven RBAC violations. Our system successfully detected all those violations and provided a report with specific locations of the violations. In this section, we will discuss how our analysis process works in detail for the mutated application.

The TMS project utilizes an annotation-based configuration technique to define application layers. REST API configurations and RBAC restrictions are also applied through annotations, which are common practice for enterprise applications. Table 2 lists frequently used annotations throughout the TMS project.

**Table 2:** Annotations used in TMS project.

Annotation	Target	Description
@Controller	3 Class	3 Indicates controller, service, and repository layers
@Service		
@Repository		
@RestController	Class	Sub type of @Controller to activate REST APIs
@RequestMapping	Class and Method	Defines HTTP types and paths for REST endpoints
@GetMapping	3 Method	3 Sub types of @RequestMapping for specific HTTP types
@PostMapping		
@DeleteMapping		
@RolesAllowed	Method	Lists a set of allowed roles

For our purpose, we only looked for the @RestController annotation in the discovery module. The HTTP and paths type were extracted from the parameters of @RequestMapping annotation or subtype annotations. Paths can be defined at both class level or method level. We aggregated the class level paths with method-level paths to get the final path for each endpoint. The endpoints’ request and response types are resolved by detecting parameters and return types of respective methods where the endpoints are defined. Finally, the RBAC roles are listed by detecting the parameters of the @RolesAllowed annotation applied to each endpoint method.

The RestTemplate class is usually used for making REST calls in the Spring Boot applications where the methods getForObject, postForObject, deleteForObject, etc. are used for performing REST calls with specific HTTP type. Each of those methods takes a URL parameter and a request object and returns a response object. We scan classes annotated with @Service annotation and filter them if they contain RestTemplate in their import statements to detect service layer REST calls. We then look for the methods described above and detect request and response types by checking the parameter type and return type. The URLs are detected by performing a backward data flow analysis recursively, as described in the proposed method section. The method calls graph is constructed by traversing each endpoint method to the service layer and repository layer methods.

**Figure 5:**
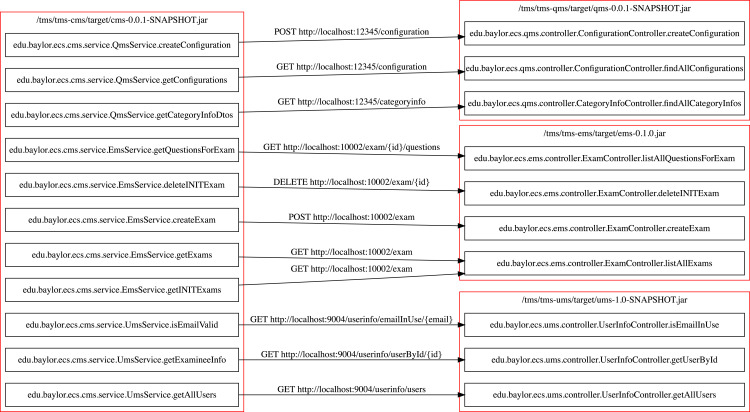
Inter microservice REST communications in TMS.

After the discovery module completes gathering metadata for each MSA, the flow matcher module combines them, and the analysis module performs the final analysis. The flow matcher module also generates a visual graph of the REST communications among the microservices using Graphviz library (Ellson et al., 2002). Figure 5 shows the generated graph for the TMS application.

While matching the request and response types, we only considered the supertype of the generic types. For example, List<AClass> and ArrayList<AClass> are considered equal during matching.

Our analyzer reported two missing-role violations for the mutated applications by specifying the fully qualified name (MSA name + package name + class name + method name) of the endpoint methods that are defined without specifying any RBAC roles. It detected two unknown-role violations along with their locations. These two violations have resulted from data entry errors where “user” and “admin” roles are mistakenly typed as “usre” and “admin” respectively, which are not present in the role hierarchy shown in Fig. 4. Our analyzer flagged one entity access violation by pointing out a pair of fully qualified method names. Methods getExams and getINITExams in CMS have the same return type List<Exam> and the same HTTP type GET but they have different RBAC roles: “user” and “moderator” respectively.

**Figure 6:**
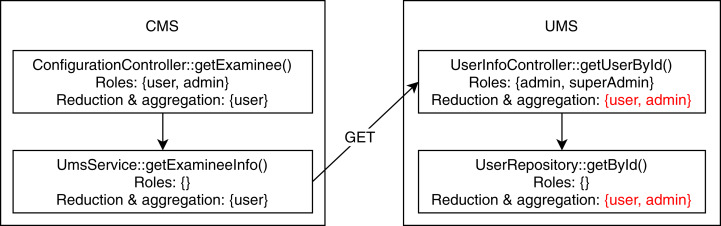
Conflicting hierarchy violation among CMS and UMS.

**Figure 7:**
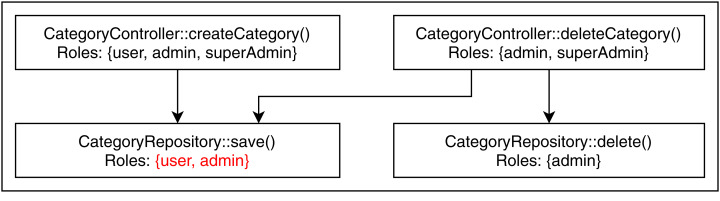
Conflicting hierarchy violation within QMS.

We found two conflicting hierarchy violations in the mutated TMS application. One of them occurred in inter microservice communication, shown in Fig. 6, where the CMS module calls the UMS module to retrieve examinee info. The getExaminee endpoint method in CMS can be accessed with a “user” role which calls the getUserById endpoint method of EMS via service layer REST call. However, the getUserById method in EMS has annotated with the “admin” role, which is a direct ancestor of the “user” role. The second conflicting hierarchy violation, shown in Fig. 7, occurred entirely within the QMS module where both createCategory and deleteCategory endpoint methods call the save method of CategoryRepository with conflicting roles. Finally, we detected one unrelated access violation between CMS and EMS, where the method getQuestions in CMS has transitive access to the method listAllQuestionsForExam in EMS via service layer REST call. They are annotated with “user” and “moderator” roles, respectively defined in separate subtrees of the role hierarchy.

We tested both source code and bytecode version of our discovery module, which utilizes the JavaParser library (Bruggen, 2020) to parse the source code and JavaAssist library (JBoss, 2020) to perform bytecode analysis to extract class definitions. We published our implementation as an open-source tool^2^ 2SAR from bytecode: https://github.com/cloudhubs/rad.^,^^3^ 3SAR from source code: https://github.com/cloudhubs/rad-source.^,^^4^ 4RBAC analysis: https://github.com/cloudhubs/rad-analysis. We ran it against the TMS project for benchmarking our analyzer and separately measured the runtime for each discovery, flow matcher, and analysis modules. For the discovery module, we break down our measurements for each microservice (CMS, QMS, EMS and UMS) and count the number of classes it scanned. Note that the discovery module performs a deep scanning for the controller layer classes that are annotated with @RestController annotation and service layer classes that have RestTemplate import to detect REST endpoints, security roles, and REST calls. For other classes, it performs just a shallow scan to construct the method call graphs.

Table 3 shows the total runtime^5^ 5The benchmark is run on a Mac OS computer with a 2.9 GHz 6-core Intel Core i9 processor and 32 GB RAM. for each module and the breakdown for the discovery module for static bytecode analysis. We can immediately see that the discovery module takes the most significant time since it performs scanning of all class files to extract metadata. In contrast, the flow-matcher and the analysis module, operating on the extracted metadata, take comparatively less time. For the discovery module, runtime depends on the number of class files in each microservices. The runtime of the flow-matcher module depends on the number of REST endpoints and the number of REST calls, while the runtime of the analysis module depends on the number of inter-microservice REST connections and the depth of the function call graph.

**Table 3:** Runtime against TMS testbed.

Module	Total	Time breakdown	
Name	Runtime (s)	MSA	Time (s)
Discovery	1.04	CMS	0.43
		EMS	0.18
		QMS	0.31
		UMS	0.12
Flow Matcher	0.13	–	
Analysis	0.29	–	

Our experiment exhibits a reasonable runtime to perform the static code analysis for enterprise applications. In total, it took 1.43 seconds against the TMS application, which consists of four microservices, a total of 102 classes, and 11 inter-microservice REST connections. For enterprise applications with many microservices, it is possible to run the discovery module in parallel for multiple microservices, which will significantly reduce the runtime.

**Figure 8:**
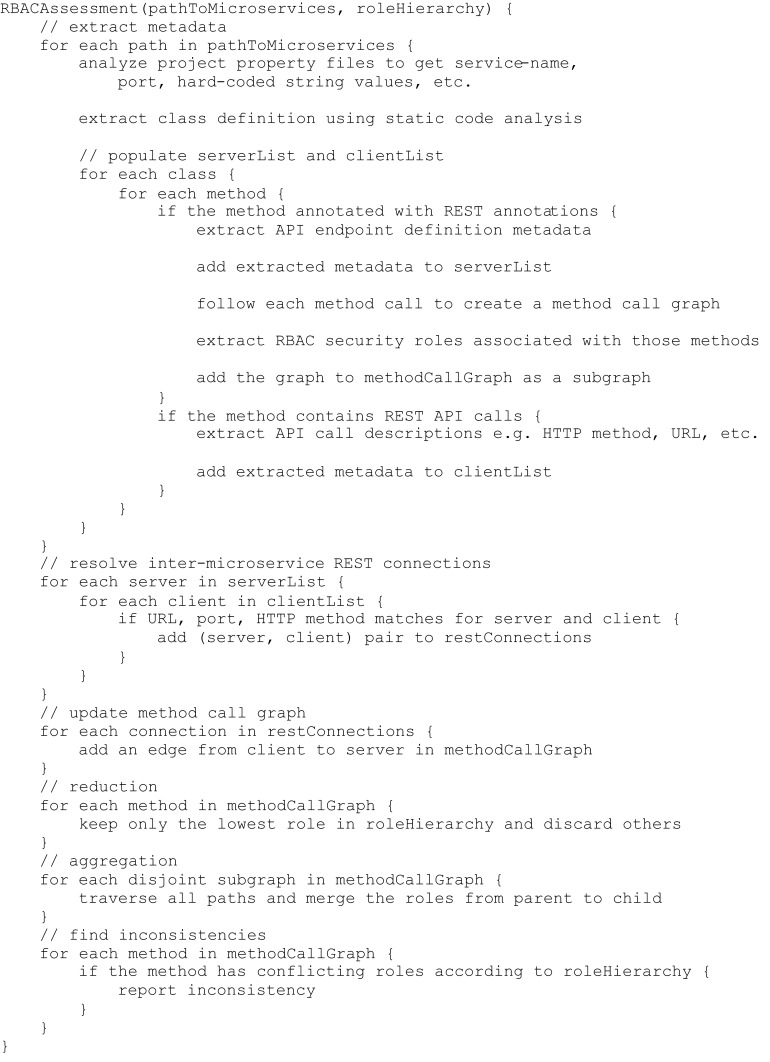
RBAC assessment pseudocode.

To show the performance of our method on larger systems, the pseudocode for our algorithm is given in Fig. 8. The amount of work necessary scales linearly with both the number of methods in the system and with the product of the REST calls and endpoints within the system, meaning our algorithm runs in *O*(*M* + *E* × *C*), where *M* is the number of methods, *E* is the number of REST endpoints, and *C* is the number of REST calls. Since the number of methods in a system is usually much larger than the number of REST calls and endpoints, our algorithm will usually run in *O*(*M*). This is in line with the results of our experiment; the discovery module, which searches every method for the needed metadata, was responsible for the majority of the time taken.

## Threats to validity

There are several threats to the validity of our work to address. Some of these arise from our experiment and some from how generalizable our approach is.

### Internal threats to validity

The primary threats to the validity of our experiment are the accuracy of the violations detected and the accuracy of the performance measures. Since we introduced known mutants for the errors, we know our tool accurately detected all of the issues. Performance-wise, we showed that our tool performed well on a small-sized application, and that the algorithm should scale up well with larger applications since the most expensive portion of the analysis scales only linearly with the number of methods in the project.

### External threats to validity

There are three external threats to our work’s validity, which may affect how generalizable our results are. First, some of the detected inconsistencies might be false positives that is, those might be intentionally left behind by the developers. Second, it depends on a user-defined role hierarchy that is assumed to contain roles universal to the application. This may not be true if users are defined in separate security realms; a role name in one realm may not be equivalent to the same role name in a different realm, either in its own access rights or in its relative position in the role hierarchy. In this case, a mapping would have to be supplied, showing which, if any, roles should be equivalent across the different realms. Another limitation is the use of security annotations. If security policies are implemented differently than through annotations, are defined in a language or a framework that does not support annotations, the current approach would not detect the roles. However, if another method was used to extract allowed roles, they could be used in the rest of the analysis process.

## Conclusion

We introduced a novel solution to automatically detect authorization inconsistencies in the RBAC implementation for enterprise applications using automated SAR. Our solution categorizes the violations into five types: missing-role violation, unknown access violation, entity access violation, unrelated access violation, and conflicting hierarchy violation. Our analyzer scans a set of microservice artifacts and provides a report listing all the possible violations by pinpointing their locations and types. While some of the detected violations may be false-positive, the violation type, along with a specific location, helps the developer easily debug them, fix them, or discard them if they were intentional. Although our analyzer was developed for a JAVA enterprise application, our proposed approach is not restricted to any particular programming language or framework. It can easily be implemented for other languages and frameworks since all modern languages now have a well-structured abstraction for REST APIs and RBAC policies.

One major shortcoming of our method is that it assumes the role hierarchy and association of users with roles are defined centrally. However, individual microservices can have separate role hierarchies or even different user-role associations. Similarly, the trust management can be distributed across multiple domains like the dRBAC. In the future, we will extend our system to address these issues to allow multiple role hierarchies and multiple role mappings along with their decentralization. Besides, we like to experiment on role assignment within a user session to identify possible inconsistencies while enforcing DSD. Our long term goal is to perform such analysis within the cloud-native environment commonly used in production deployments, for example, analyzing Dockerfiles and Kubernetes artifacts.

## Additional Information and Declarations

### Data Availability

The following information was supplied regarding data availability: Data and analysis are available at GitHub, specifically: - Bytecode analysis: https://github.com/cloudhubs/rad. - Source code analysis: https://github.com/cloudhubs/rad-source. - RBAC analysis: https://github.com/cloudhubs/rad-analysis.
